# Plasma Leak From the Circulation Contributes to Poor Outcomes for Preterm Infants: A Working Hypothesis

**DOI:** 10.3389/fneur.2021.636740

**Published:** 2021-08-02

**Authors:** Yvonne A. Eiby, Barbara E. Lingwood, Ian M. R. Wright

**Affiliations:** ^1^Faculty of Medicine, Perinatal Research Centre, Centre for Clinical Research, The University of Queensland, Brisbane, QLD, Australia; ^2^Department of Neonatology, Royal Brisbane and Women's Hospital, Brisbane, QLD, Australia; ^3^The School of Medicine, Illawarra Health and Medical Research Institute, University of Wollongong, Wollongong, NSW, Australia; ^4^Australian Institute of Tropical Health and Medicine, The College of Medicine and Dentistry, James Cook University, Cairns, QLD, Australia

**Keywords:** hypovolemia, blood pressure, premature infant, volume expansion, cerebral blood flow, brain oxygenation, blood volume, preterm infants

## Abstract

Preterm infants are at high risk of death and disability resulting from brain injury. Impaired cardiovascular function leading to poor cerebral oxygenation is a significant contributor to these adverse outcomes, but current therapeutic approaches have failed to improve outcome. We have re-examined existing evidence regarding hypovolemia and have concluded that in the preterm infant loss of plasma from the circulation results in hypovolemia; and that this is a significant driver of cardiovascular instability and thus poor cerebral oxygenation. High capillary permeability, altered hydrostatic and oncotic pressure gradients, and reduced lymphatic return all combine to increase net loss of plasma from the circulation at the capillary. Evidence is presented that early hypovolemia occurs in preterm infants, and that capillary permeability and pressure gradients all change in a way that promotes rapid plasma loss at the capillary. Impaired lymph flow, inflammation and some current treatment strategies may further exacerbate this plasma loss. A framework for testing this hypothesis is presented. Understanding these mechanisms opens the way to novel treatment strategies to support cardiovascular function and cerebral oxygenation, to replace current therapies, which have been shown not to change outcomes.

## Introduction

Preterm infants are at high risk of poor neurodevelopmental outcome. Survival rates of preterm infants have improved with modern care, such that in a recent study across 10 neonatal networks 87% of infants born at 24–30 wk survived (1). However, these improvements in survival have not resulted in a proportional reduction in rates of disability. In preterm infants born at 24–26 weeks in the US, UK and Sweden, 25–40% of survivors have a moderate to severe disability (2). The reasons for impaired brain development are likely multifaceted and include infection (pre and post-natal), cardiopulmonary immaturity contributing to global hypoxia/ischemia and intra-ventricular hemorrhage. However, continuing occurrence of adverse outcomes in preterm infants indicates that some causes and potential interventions remain to be understood.

It is uncertain whether oxygen delivery is adequate during the first days of life. Healthy preterm infants have resting cerebral blood flows averaging 15 mL/100 g/min (range 7–25 mL/100 g/min), this value is 30% lower for ventilated preterm infants (10 mL/100 g/min) whereas, adult values (45–50 mL/100 g/min) are 3–4.5 x higher (3). However, these global measures of flow may mask the vulnerability of white matter as arterial end zones of periventricular white matter are more sensitive to low perfusion pressure than the brain as a whole (3). This selective vulnerability to ischaemia during hypotension means that preterm infants with a blood pressure < 29–30 mmHg, identified by several studies as the threshold below which cerebral autoregulation fails, are likely to have lower flow to white matter than global measures predict (4). But caution is necessary when applying this single blood pressure threshold as maturation-dependent differences in mean blood pressures are observed clinically, such that mean blood pressures increase with gestational age (5). In addition to low perfusion, hypoxemia may occur in individuals with shunts due to delivery of poorly oxygenated blood to the brain (6). Evidence of low cerebral tissue oxygenation, elevated oxygen extraction or low venous oxygen saturation, suggest that in some preterm infants, oxygen extraction is approaching the limit of supply leaving these infants at risk of brain injury (6–8). Infants <1,250 g who died or had severe neuroradiographic abnormalities had significantly lower average cerebral oxygen saturation than infants that survived without abnormalities (67 vs. 72%; *p* = 0.02) (8). Preterm infants with elevated cerebral oxygen extraction (cFTOE > 0.4) had increased risk of death or brain injury (7). In a small group of preterm infants, cerebral venous saturation was 55% (3). In adults and term infants, cerebral venous oxygen saturation is about 65%, and it has been suggested that the lower values observed in preterm infants may indicate inadequate supply (3). Together these findings support the concept that cardiopulmonary immaturity is a significant contributor to poor neurodevelopmental outcomes in preterm infants.

The traditional approach to supporting cardiovascular function and protecting the brain in preterm infants has focussed on cardiac performance. Standard treatments include crystalloid volume expansion and inotropic support, usually with dopamine or dobutamine. Cochrane reviews indicate that neither treatment has improved long-term neurodevelopmental outcomes (9, 10). Furthermore, there is mounting evidence that both approaches may be associated with poorer outcomes. Extremely preterm infants exposed to antihypotensive therapies had an increased risk of death/neurodevelopmental impairment (OR 1.836, 95% CI 0.709–3.307) even when initial markers of illness were accounted for in the statistical model (11). Despite this, these approaches still form the mainstay of cardiovascular support in clinical practice in preterm infants, as there is no effective alternative approach to fill this therapeutic void. There is a critical need for more effective approaches to cardiovascular support and therefore reduction in brain injury in these very preterm infants. The purpose of this paper is to stimulate discussion about contributors to cardiovascular instability and potential new interventions to address these contributors.

Existing treatment strategies have largely ignored a whole area of cardiovascular physiology – loss of plasma from the circulation, occurring at the capillaries. We propose that specific preterm vulnerabilities at this level lead to hypovolemia, subsequent hypotension and/or low cardiac output and ultimately inadequate cerebral blood flow.

## Our Hypothesis

We propose that, in the preterm infant, high capillary permeability, altered hydrostatic and oncotic pressure gradients, and reduced lymphatic return all combine to increase net loss of plasma from the circulation, resulting in hypovolemia; and that this is a significant driver of cardiovascular instability and poor cerebral oxygenation.

### An Old Paradigm Revisited – Hypovolemia Does Occur in PreTerm Infants Soon After Birth

Current belief is that hypovolemia is not a significant contributor to mortality or morbidity in preterm infants. This conclusion is largely based on the ineffectiveness of crystalloid solution for improving outcomes (10). It is assumed that if volume expansion (predominantly with saline) does not improve outcomes, then hypovolemia is not a significant contributor to poor outcomes. We propose that failure of volume expansion with saline does not indicate absence of hypovolemia but is due to the inherent characteristics of the preterm circulation. The capillaries in the preterm infant are significantly more permeable than in the adult, such that saline is retained within the circulation for a very short time (85% lost within 10 min) (12–14) and thus effective volume expansion cannot be achieved with saline. In addition, administration of excessive volumes of saline may lead to accumulation of fluid within the tissue, acidosis and poor outcomes (15–17). Likewise, studies of colloids for volume expansion in preterm infants have failed to improve acute cardiovascular function or long-term outcomes (18–20) as colloids are not effective at increasing blood volume. As for saline, colloids are rapidly lost from the circulation. This is evidenced by a lack of sustained increase in oncotic pressure and/or protein concentration following colloid infusion (20, 21). A similar lack of effect is observed in sick adults where an increase in capillary permeability is cited as a potential cause (22). These studies indicate that permeability of preterm capillaries is so high it allows plasma proteins to rapidly leak out of the circulation. So failure of crystalloids or colloids to improve outcomes is due to their ineffectiveness at increasing blood volume rather than the absence of hypovolemia.

### Evidence for Hypovolemia in PreTerm Infants

Early measures of blood volume relied on labeled plasma and dyes and reported higher average values for preterm compared to term infants (83–109 vs. 75–99 mL/kg across multiple studies) (23–26). As a result, current guidelines estimate blood volume to be greater in preterm than term babies, for example 100 vs. 80 mL/kg (27) or 90–95 vs. 80–85 ml/kg (28) (for both preterm vs. term infants, respectively). However, it has been suggested that these early volume measurements in preterm infants have provided erroneous results due to rapid loss of the small tracers from the circulation (29). More recent studies using larger tracers, have suggested lower blood volumes for preterm infants than previously reported (68.6, 77.9, 72.3, and 71 mL/kg) (29–32). Importantly, the range of blood volumes reported in both these old and newer studies are highly variable, for example 55–134, 46–131, 49–119, 53–105, 45–103 mL/kg. It is possible that babies at the lower end of these ranges are hypovolemic. These low blood volumes may be due to loss of plasma from the circulation. Even in term babies where clinical care has altered less than in preterm babies, large shifts in plasma volume soon after birth have been reported (25, 26, 33).

Echocardiographic evidence of hypovolemia is lacking but this may be due to technical limitations rather than absence of hypovolemia. Even in adults, no echo parameter has been shown to be a reliable predictor of measured hypovolemia (34). We are not aware of any studies that have compared direct measures of blood volume with echo parameters such as atrial and IVC dimensions in neonates.

Studies of delayed cord clamping support early plasma loss. Even after delayed cord clamping, 16% of preterm infants (<32 wk) have a blood volume lower than 63 mL/kg (30). Earlier studies measuring blood volume after DCC show that much of the initial blood volume increase is lost within the first 4 h after birth (35, 36) as predicted by our hypothesis. The resultant increased red cell mass (37) would explain why cerebral hemoglobin concentration is increased but may not be sufficient to counteract the volume effects of plasma loss.

**Figure 1:**
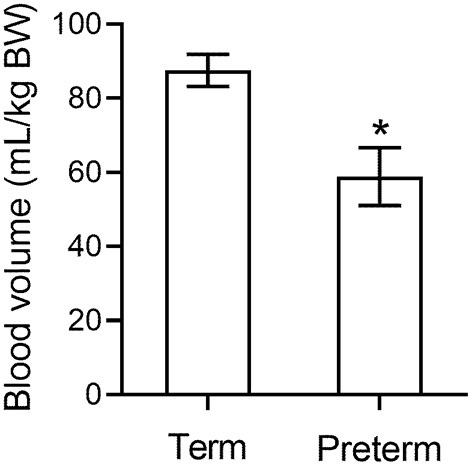
Blood volume in preterm piglets (*n* = 26) is significantly lower than in term piglets (*n* = 3) (39). * indicates *p* < 0.001.

**Figure 2:**
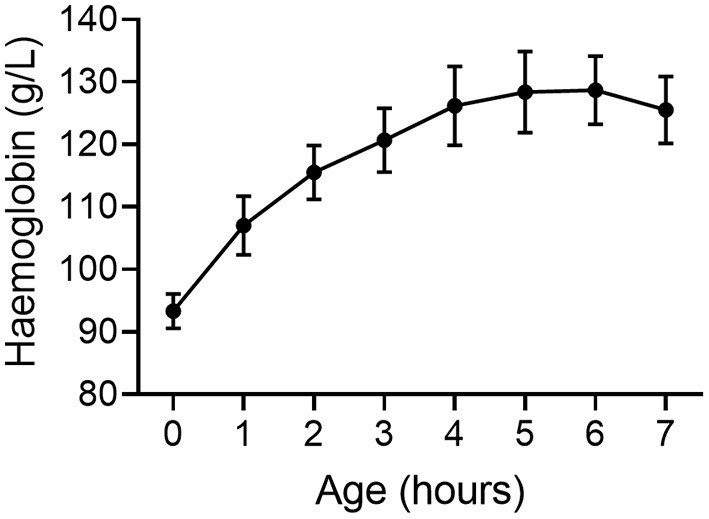
Hemoglobin concentration increases following birth in very preterm piglets (*n* = 10) suggesting a loss of plasma from the circulation and a reduction in blood volume (39). Mean ± SEM.

We have conducted extensive studies in preterm piglets at 98/115 d gestation where respiratory requirements, cardiovascular instability, thermoregulation and other factors are similar to a human neonate born at 27 wk gestation (38). We measured blood volume using a large tracer that does not rapidly leak from the circulation. Blood volume 5 h after birth in preterm piglets is significantly lower than in spontaneously born term piglets at 18 h old (*p* < 0.001, Figure 1) (39) further supporting that hypovolemia is already present by this time. Parallel data in the same animals indicates an increase in hemoglobin levels in the 6 h after birth (Figure 2) suggesting that the low blood volume observed is due to loss of plasma. There is limited data describing changes in hemoglobin levels in human infants in the absence of externally administered fluids however, similar fluid shifts to those seen in the preterm piglet can not be excluded. Our discussions below describe a hypothesis that a similar loss of plasma volume may contribute to poor neurodevelopmental outcomes in some preterm infants.

### Consequences of Hypovolemia

We have already demonstrated that preterm piglets are extremely sensitive to low preload and hypovolemia, and unable to maintain blood pressure or cerebral blood flow when blood volume is reduced (40, 41). This is in contrast to term piglets where removal of similar blood volumes/kg also results in reduced blood pressure but not reduced cerebral blood flow (40). These differing responses may be due to immaturity of neurohumoral compensatory systems such as sympathetic and adrenergic control of the circulation, RAAS and the baroreflex (42–48). Thus, hypovolemia in preterm infants due to rapid early loss of plasma may contribute to adverse preterm outcomes.

## What are the Determinants of Plasma Loss at the Capillary?

**Figure 3:**
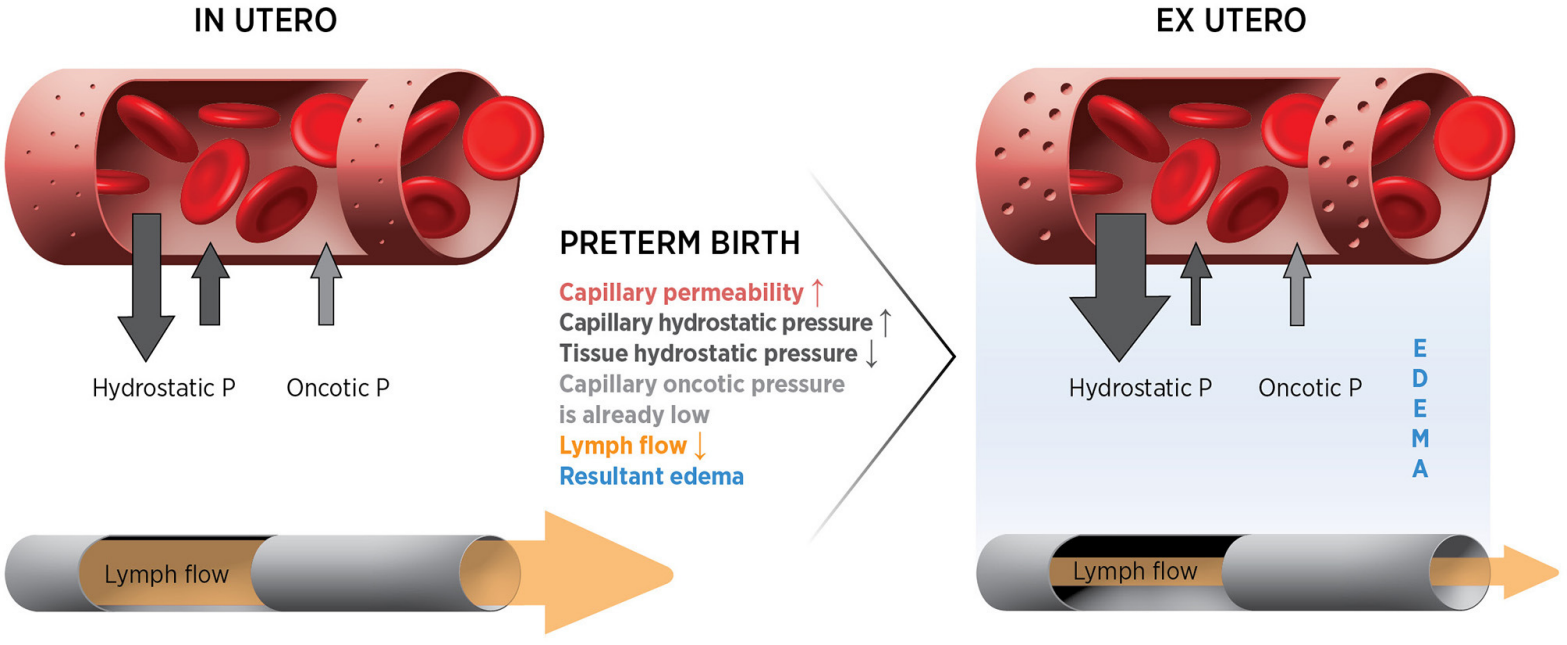
Forces and potential changes affecting fluid movements at the capillary after preterm birth.

The net movement and type of fluid leaving capillaries is determined by biophysical forces, structural elements and biological moderators, such as inflammation. Multiple biophysical forces affect movement of fluid in or out of the capillary and these are described by the Starling equation:

$$\begin{array}{l} \text{J}\text{v}/\text{A}\;=\text{L}\text{p}\;\left(\left({\text{P}}_{\text{c}}-{\text{P}}_{\text{i}}\right)-\sigma \;\left(\pi_{\text{p}\;}-\;\pi_{\text{i}}\right)\right) \end{array}$$

where Jv/A = filtration rate/unit area; Lp = filtration co-efficient; P = hydrostatic pressure in capillary or interstitial tissue; σ = reflection co-efficient; π = oncotic pressure of plasma or interstitial fluid. Thus, filtration is determined by capillary permeability in combination with hydrostatic and oncotic pressure gradients. Loss from the circulation is also determined by the rate at which fluid in the tissue is returned to the circulation via the lymphatic vessels. In the fetus, loss from the capillaries is very high but is matched by very high lymphatic return (49, 50). As a result, plasma volume remains stable. Lymph flow may be operating near capacity, resulting in a system that is close to tipping point. Thus, even small changes in the system (e.g., increased capillary permeability, low oncotic pressure, increased hydrostatic pressure gradient or reduced lymph flow) will result in loss of plasma from the circulation (Figure 3). Thus, we hypothesis that the classic Starling equilibrium is thus uniquely disturbed in the preterm infant with the resulting disequilibrium leading to hypovolemia.

### Capillary Permeability Is High in PreTerm Infants and Increases With Inflammation

Capillary permeability decreases with developmental maturity. Estimation of blood volume using indocyanine green in human infants consistently reports higher values than hemoglobin subtype dilution, suggesting rapid leakage of the dye out of the vascular space (29). Turnover of albumin from the circulation is 18.4 ± 6.8%/h in term babies (51, 52) which is 3–4 times faster than the 5%/h reported in healthy adults (26, 51). This rapid turnover of albumin indicates a higher rate of capillary leak. In the sheep fetus, intravascular loss of infused saline is 93–94% over the first hour compared to 60–70% in the term newborn (12, 13). In sheep, the fetal whole body capillary filtration coefficient may be 5–6 times that of the adult (14). These values are supported by measures of lymphatic flow that are markedly higher in lambs compared to adults and are higher still in the fetus (49, 50). These very high filtration rates are driven, at least partly, by high capillary permeability.

There is very limited information regarding capillary structure and ultrastructure in preterm infants and how this may contribute to capillary leak and plasma volume loss. Many vessels in the rapidly growing organism are in an angiogenic state and these immature vessels will therefore be more leaky (53). Ultrastructure changes are thought to contribute to capillary leak in adults with conditions such as sepsis or tumors (53–55). Studies assessing capillary ultrastructure in preterm neonates are lacking but whole-body rapid growth necessitates vasculogenesis so it is likely that various aspects of capillary ultrastructure are immature in preterm infants. Investigations of the development of critical structural components such as the calyx, pericytes and gap junctions and these are an essential component to understanding preterm blood volume changes after birth.

Inflammation exacerbates this trans-capillary leak (56). In older children and adults both sepsis and the resultant inflammation contribute to leak as part of septic shock (55). Preterm infants have a vigorous inflammatory response compared to older babies and have a limited ability to produce anti-inflammatory mediators (57). There is an inverse association between gestation (23–28 wk) and blood concentrations of numerous inflammation-related proteins, even on day 1 of post-natal life and in the absence of pre-existing inflammation (57). Other neonatal conditions, such as hypoxic ischemic encephalopathy, are also associated with both increased inflammatory response and capillary leak within the brain parenchyma. It is thus well-established that inflammation causes an increase in capillary permeability at all ages (14). Mathematical modeling suggests the increases in capillary permeability that may occur during mild inflammation would result in up to 5x the rate of plasma loss (58). Therefore, our hypothesis is that in some preterm infants systemic inflammation is contributing to excessive fluid movement into the tissue in the first hours of life.

### The Oncotic Pressure Gradient Is Decreased in PreTerm Infants

The oncotic pressure gradient across the capillary membrane is generated by the presence of large protein molecules in the capillary and opposes movement of fluid out of the capillary (Figure 3). Preterm infants have low plasma protein levels (19 g/l at 26 wk compared to 31 g/L at term) (59), as do our preterm piglets (25 g/L compared to 48 g/L at term). Low plasma protein levels in preterm infants may limit the trans-capillary oncotic gradient favoring movement of fluid out of capillaries. Numerous clinical studies have shown an association of low plasma protein levels in preterm infants with poor cardiovascular function and adverse outcomes (60, 61). At 24 h of life, very preterm infants with low plasma protein levels had lower blood pressure and longer capillary refill times compared to those with normal plasma protein levels, even after adjusting for gestational age (both *p* < 0.001) (60). Our hypothesis predicts that adverse outcomes in those with low protein levels may be due to the low oncotic pressure gradient contributing to plasma loss and hypovolemia.

### The Hydrostatic Pressure Gradient Across the Capillary Increase After Birth

An increase in the hydrostatic pressure gradient across a capillary will increase fluid loss (Figure 3). It is unknown whether the hydrostatic pressure gradient increases after birth in preterm infants as measures of tissue pressure and capillary pressure are not feasible. Some reports suggest that the transition from a fetal to neonatal circulation is associated with an increase in blood pressure (62, 63), while others suggest little change at birth (64). It is also unclear if increases in arterial pressure lead to increases in capillary hydrostatic pressure. There may be a decrease in tissue hydrostatic pressure at birth due to removal of the externally applied intra-amniotic fluid pressure of 1–8 mmHg (65). Additionally, the initial high transcutaneous fluid loss that occurs after birth in very preterm infants may act to maintain a low tissue hydrostatic pressure (66). So the hydrostatic pressure gradient between the capillary and the tissue may increase even if capillary pressure is unchanged. Whether this combination of pressure changes will increase the trans-capillary pressure gradient and favor fluid movement out of the circulation into the tissue needs to be addressed in an animal model. Adult mathematical modeling based on the Starling equation of fluid movement suggests that even small changes in blood pressure could result in significant movement of fluid out of the vasculature (58).

When inotropes (or indeed any treatment) are used to increase blood pressure in preterm infants, the increase in driving pressure may increase the hydrostatic pressure gradient across the capillary. This may actually exacerbate fluid loss according the Starling equation (Figure 3). Our preterm piglets treated with dopamine had a modest increase in blood pressure, as do preterm infants treated with dopamine. The blood pressure after treatment ended was 7 mmHg below pre-treatment levels. This reduction in blood pressure may be due to an increase in driving pressure produced by dopamine leading to an increase in the hydrostatic pressure gradient across the capillary and thus plasma loss. It needs to be determined if this partly explains why dopamine and dobutamine are ineffective for improving outcomes (9) and why “permissive hypotension” does not result in worse outcomes (67).

### Lymphatic Flow May Be Insufficient to Match Plasma Filtration

The interstitial tissue is capable of holding a large fluid volume, as demonstrated by the clinical condition of fetal hydrops. If this fluid is not returned to the circulation, hypovolemia will result. Downstream capillaries are no longer considered to be in a state of sustained reabsorption (68) so fluid balance is critically dependent on adequate lymphatic return. Lymphatic flows in fetal sheep are 2.5–5x higher than in adult sheep (49, 50). This high flow rate in the fetus is adequate to deal with high capillary filtration rates *in utero*. However, it is unclear whether preterm infants may be operating at the upper limit of lymphatic return with little reserve capacity to compensate for the increases in filtration after birth. Indeed, the lymphatic structure and function of preterm infants is largely unknown and warrants investigation.

Iatrogenic factors may further compromise lymphatic return in the *ex utero* preterm. Lymphatic return in fetal sheep is extremely sensitive to increases in intrathoracic pressure which impede flow. Positive pressure support increases intrathoracic pressure and may thus also impede lymph flow, indeed it may cease altogether at intrathoracic pressures only marginally above normal (49). This may, along with increased cardiac output due to improved venous return, contribute to reduction in cardiovascular support required by preterm neonates with the reduced mean airway pressures used in continuous positive pressure rather than ventilator driven support (69, 70). Systemic inflammation, which is common in preterm infants (57), has also been shown to negatively influence lymphatic contraction which is critical to maintaining high flow rates particularly in an immobile population (71). So animal experiments designed to test if either elevated intrathoracic pressure or inflammation limits lymphatic flow are necessary to determine if tissue clearance of fluid is compromised.

## Discussion

The evidence presented supports our hypothesis that preterm infants are at high risk of mortality and morbidity because of a unique conjunction of features in the small vessels that make up the majority of the circulatory system. These include that:

- Capillary permeability increases with prematurity, and with inflammation;
- Low plasma protein levels in preterm neonates result in reduced oncotic pressure gradients at the capillary;
- Capillary hydrostatic pressure gradients are increased postnatally in preterm neonates;
- Preterm neonates have impaired postnatal lymphatic flow.

All of these factors would contribute to plasma volume loss resulting in hypotension, and reduced cerebral blood flow and oxygenation in preterm infants. However, it is unclear which factor/s have the greatest influence on plasma loss from the circulation. To test our hypothesis we are undertaking experiments that will provide quantitative assessment of each factor that could drive plasma loss from the circulation and factors that may reduce lymphatic flow (Table 1). These experiments also allow evaluation of resultant edema, hypovolemia, hypotension, and poor cerebral oxygenation. Using preterm piglets delivered when developmentally similar to preterm infants born at 27 wk gestation and cared for using standard intensive care techniques, we are able to observe changes in cardiovascular and lymphatic physiology over the first 12 h of life, when cardiovascular instability typically occurs in preterm infants.

**Table 1:** Experiments in preterm and term piglets to test our working hypothesis.

**Aspects of hypothesis to test**	**Data required**
Capillary permeability is increased in preterm neonates	Measure permeability as the rate that different sized molecules are lost from the circulation in preterm and term neonates. This will also determine the size required for effective volume expansion.
Capillary permeability is increased with inflammation	Measure the association between inflammation levels and the change in permeability over the 12 h after birth.
Low oncotic pressure gradients are associated with loss of plasma from the circulation	Measure plasma and tissue protein concentrations (oncotic pressure gradient). Is this associated with the change in plasma volume between birth and 12 h old?
Increased capillary hydrostatic pressure gradient in preterm neonates	Estimate hydrostatic pressure gradient by measuring the difference between blood pressure and tissue pressure. Does this correlate with changes in plasma volume?
Reduced lymphatic flow	Does lymphatic flow decrease across the 12 h after birth?
Lymphatic flow is impaired by inflammation	Measure the association between inflammation levels and lymphatic flow across the first 12 h of life.
Lymphatic flow is impaired by high intrathoracic pressure	Measure lymphatic flow during low and high intrathoracic pressure (ventilation induced mean airway pressure).
High plasma loss from the circulation results in edema, hypotension and low cerebral oxygenation	Association between change in plasma/blood volume and edema (tissue water content), blood pressure and cerebral brain oxygenation.
Priority treatment targets	From above experiments, quantitative changes in factors will be used to model the influence of each factor on plasma loss from the circulation.

This quantitative data can be used to create a mathematical model of the Starling equation specific to the preterm infant. By expanding this model to include lymphatic return and blood volume we can measure the relative contribution of each factor to identify priority targets for new treatments to effectively support preterm cardiovascular function. Subsequent experiments would then test interventions targeting critical factors identified by the modeling.

1. If high capillary permeability is a major contributor to plasma loss then studies would aim to develop volume expanders large enough to stay within the circulation. Albumin sized volume expanders are not suitable because these leak from the circulation (39) and potentially cause adverse effects. Volume expanders based on larger molecules that remain within the vascular space are likely to be more effective but their use must be tempered by the associated risk. Our proposed permeability studies with different sized molecules will determine the size required for effective volume expansion. We have shown that red blood cell transfusion is effective for increasing blood volume in preterm piglets (72). Early (within 12 h of birth) transfusions as proposed for treatment of anemia of prematurity (73) and could provide an effective form of volume expansion. Understanding the developmental structural changes occurring in the capillaries that underlie these functional changes will identify further possibilities for therapeutic interventions that have not been considered in this vulnerable group. Other more novel target pathways that are known to moderate vascular leak would include targeting Vascular Endothelial Growth Factor (VEGF) mediated leak or modifying the effects of local gasotransmitter-mediated (Nitric oxide, Hydrogen sulfide) capillary leak (74), but appropriate animal and then human studies will be required.
2. If the hydrostatic pressure gradient across the capillary is a major contributor to plasma loss, then studies of the effects of blood pressure targets, “permissive hypotension” and of various inotropic interventions on plasma loss could be conducted. The exact point at which raising the blood pressure is likely to be effective rather than counterproductive is not known (11, 67, 75), nor is the effect of constrictive vs. dilatory inotropes. It is likely to vary by gestation, post-natal age and individual circumstances and that blanket algorithms are not serving our varied clinical populations.
3. Our studies will indicate if decreased lymphatic flow is a major contributor to plasma loss and whether this is due to increased intrathoracic pressure or inflammation. If high intrathoracic pressure is the critical factor then this would support the continued use of lower intrathoracic pressure ventilation strategies. If inflammation is limiting lymphatic flow then there are a number of pharmacological approaches that may be beneficial including corticosteroids or other anti-inflammatory agents, acting to improve lymphatic contraction and potentially also decrease capillary permeability. There is some evidence that administration of low-dose hydrocortisone at 12–48 h after birth results in decreased mortality (76). These studies would indicate if administration needs to occur at birth in order to prevent early plasma loss. To our knowledge treatment this early has never been trialed.

In summary we propose that a major driver of cardiovascular compromise in the preterm infant is occurring at the level of the microcirculation with a net volume loss that exceeds the ability to compensate. This may lead to both increased mortality and long-term disability and is an urgent area for further research to improve preterm outcomes.

## Data Availability Statement

The raw data supporting the conclusions of this article will be made available by the authors, without undue reservation.

## Ethics Statement

The animal study was reviewed and approved by Health Sciences Animal Ethics Committee, The University of Queensland.

## Author Contributions

All authors contributed to conception and design, statistical analysis and interpretation, wrote and revised the manuscript and approved the submitted version.

## Conflict of Interest

The authors declare that the research was conducted in the absence of any commercial or financial relationships that could be construed as a potential conflict of interest.

## Publisher's Note

All claims expressed in this article are solely those of the authors and do not necessarily represent those of their affiliated organizations, or those of the publisher, the editors and the reviewers. Any product that may be evaluated in this article, or claim that may be made by its manufacturer, is not guaranteed or endorsed by the publisher.
